# Estimation of Parameters Influencing Waterborne Transmission of Infectious Hematopoietic Necrosis Virus (IHNV) in Atlantic Salmon (*Salmo salar*)

**DOI:** 10.1371/journal.pone.0082296

**Published:** 2013-12-05

**Authors:** Kyle A Garver, Amelia A. M. Mahony, Dario Stucchi, Jon Richard, Cecile Van Woensel, Mike Foreman

**Affiliations:** 1 Pacific Biological Station, Fisheries and Oceans Canada, Nanaimo, British Columbia, Canada; 2 Institute of Ocean Sciences, Fisheries and Oceans Canada, Sidney, British Columbia, Canada; INRA France

## Abstract

Understanding how pathogenic organisms spread in the environment is crucial for the management of disease, yet knowledge of propagule dispersal and transmission in aquatic environments is limited. We conducted empirical studies using the aquatic virus, infectious hematopoietic necrosis virus (IHNV), to quantify infectious dose, shedding capacity, and virus destruction rates in order to better understand the transmission of IHN virus among Atlantic salmon marine net-pen aquaculture. Transmission of virus and subsequent mortality in Atlantic salmon post-smolts was initiated with as low as 10 plaque forming units (pfu) ml^−1^. Virus shedding from IHNV infected Atlantic salmon was detected before the onset of visible signs of disease with peak shed rates averaging 3.2×10^7^ pfu fish^−1^ hour^−1^ one to two days prior to mortality. Once shed into the marine environment, the abundance of free IHNV is modulated by sunlight (UV A and B) and the growth of natural biota present in the seawater. Virus decayed very slowly in sterilized seawater while rates as high as k =  4.37 d^−1^ were observed in natural seawater. Decay rates were further accelerated when exposed to sunlight with virus infectivity reduced by six orders of magnitude within 3 hours of full sunlight exposure. Coupling the IHNV transmission parameter estimates determined here with physical water circulation models, will increase the understanding of IHNV dispersal and provide accurate geospatial predictions of risk for IHNV transmission from marine salmon sites.

## Introduction

Infectious hematopoietic necrosis virus (IHNV) is an aquatic rhabdovirus that causes devastating epizootics in salmon and trout species in the Western N. America, Europe, and Asia. With the appropriate virus strain, host condition, and environmental parameters, IHNV can infect and cause an acute systemic disease in all five species of Pacific salmon, Atlantic salmon, and rainbow trout [1]. In the northern reaches of the IHNV geographical range encompassing Alaska, British Columbia, and Kamchatka Peninsula, the virus predominately infects sockeye salmon [2], [3], [4].

In British Columbia, IHNV was first identified from sockeye salmon in 1959 at Cultus Lake [5] however from earlier descriptions of annual epizootics at a sockeye salmon hatchery on the Fraser River [6] it is speculated that IHNV was likely present since at least 1925 [7]. The virus has also been associated with mortality events of Atlantic salmon cultured in the marine waters of British Columbia. Since the introduction of Atlantic salmon to the BC coast in the mid 1980’s, there have been multiple events whereby IHNV was detected in net-pen Atlantic salmon. The first occurrence was in 1992 farmed Atlantic salmon in sea water net-pen sites off the east coast of Vancouver Island. During this initial outbreak, which spanned 4 years (1992–1996), the virus spread to 13 additional sites within 11-nautical-mile radius of the index case resulting in farm level mortality up to 78% [8]. In a second outbreak, lasting from 2001–2003, IHN disease was even more widespread with a total of 36 Atlantic salmon farms contracting the virus [9]. Most recently, in 2012, the virus was again detected in three separate Atlantic farm sites and as with previous occurrences, devastating economic losses were incurred either as a consequence of mass mortality events or from depopulation of the site to circumvent further spread of the disease. The estimated economic loss resulting from the first two epizootics was $40 million in inventory representing $200 million in lost sales (Odd Grydeland, personal communication).

Important questions concerning outbreaks in farmed Atlantic salmon are: what is the risk of virus dispersion from an IHNV infected site and what factors influence the spread of virus? Studies investigating spatial and temporal patterns of the IHNV outbreaks suggested that farming practices, such as boat movements and the use of shared personnel and contractors contributed to farm to farm spread of disease both within and between areas [9]. Consequently in 2010, the BC salmonid aquaculture industry implemented a viral management plan which enacts strict biosecurity measures to control the anthropogenic risk factors influencing viral spread. Procedures such as the immediate quarantine of an infected farm site have proven effective at reducing the spread of disease. Nevertheless, the mechanism of viral dispersion via natural waterborne transmission cannot be discounted and has been identified as a key factor for the spread of IHN disease among Atlantic salmon net-pen sites.

Laboratory studies have clearly indicated that Atlantic salmon are susceptible to IHNV through waterborne exposure of virus [10]. Moreover IHNV survives temporarily in saltwater when held in laboratory conditions [11], [12], [13] making it plausible that viable and infective virus could be transmitted by water movement (i.e. ocean currents) from IHNV infected Atlantic salmon farms to uninfected fish. However to assess the risk of viral dispersion from an infected farm site requires knowledge of the infection dynamics of IHNV in Atlantic salmon. Specifically, the key parameters required in assessing waterborne transmission risk are the relationship between the rate of IHNV shedding from infected Atlantic salmon, the stability of virus in sea water and the minimum virus dose required to induce IHN disease in Atlantic salmon. Thus, it was the aim of this study to determine quantitative estimates of these waterborne transmission parameters associated with IHNV infection of Atlantic salmon.

## Materials and Methods

### IHN virus amplification and quantification

**Figure 1:**
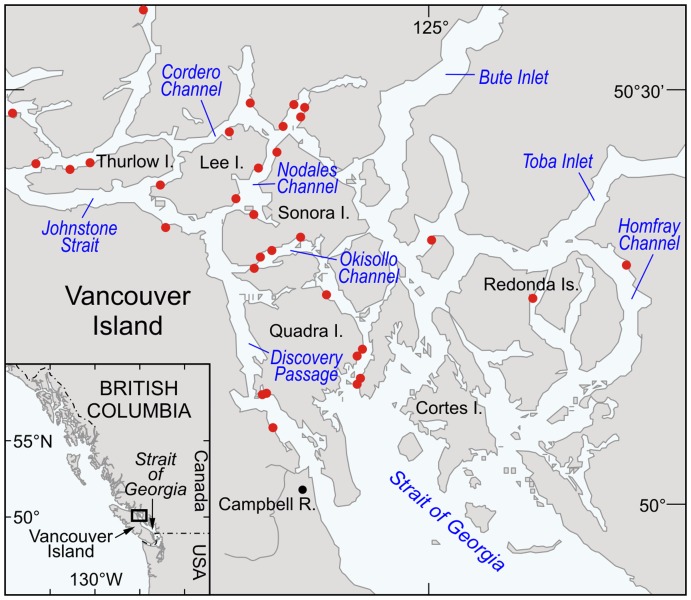
Map depicting Discovery Islands, British Columbia, Canada, with location of salmon aquaculture sites marked by filled (red) circles.

The virus isolate, BC93-057, was used for all stability and challenge trials described herein and originates from pen-reared Atlantic salmon (*Salmo salar*) from Discovery Islands, British Columbia in 1993 (Fig 1). BC 93-057 groups into the U genogroup and is representative of the virus type associated with the epizootic events of 1992-1996 that occurred in the Atlantic salmon aquaculture industry in the marine waters off the East Coast of Vancouver Island, BC. Virus stock with low passage number (<3) was obtained by inoculating a monolayer of *Epithelioma papulosum cyprini* (EPC) cells [14] [15] at a multiplicity of infection of 0.001. Upon observation of total cytopathic effect, the cell culture was harvested, centrifuged at 3000 g for 15 min, and stored in various volumes at –80°C. Upon thawing, the virus stock titer was determined by plaque assay using EPC monolayers pretreated with 7% polyethylene glycol [16]. Cell culture plates were incubated for 5–7 days to allow for cytopathic effects. Cells were fixed with 10% formalin and stained with a 0.1% crystal violet solution and plaques were enumerated and reported as plaque forming units per ml (pfu ml^−1^).

### IHNV Stability studies

**Seawater parameters.** Natural seawater was taken from the Strait of Georgia at a depth of 22 metres. Collection of seawater in British Columbia does not require special permissions; therefore no permits were obtained for this field sampling. Salinity, measured using a STX-3 refractometer (VeeGee) was 31±1 ppt. An aliquot of the natural seawater was autoclaved at 121°C for 30 minutes at 15 psi (BetaStar) to generate sterile seawater.

**Viral decay in the absence of sunlight.** Natural seawater samples (30 ml) contained in sterile, capped (denoted as closed) or uncapped (denoted as open) 50 ml tubes were seeded with 3×10^6^ pfu of IHNV (isolate 93-057) following a 30 minute acclimation to either 8, 10, or 12°C. Fetal bovine serum (0.08%) was present in the seeded sample due to carry over from cell culture media present in the virus stock. Consequently, the stability estimates obtained herein may be conservative due to the enhanced stability afforded by low levels of serum. Temperatures were chosen to encompass the annual temperature range recorded from an Atlantic salmon farm site in Okisollo Channel of the Discovery Islands, British Columbia, Canada (Fig 1). The ‘open’ and ‘closed’ virus spiked seawater samples were incubated in the dark at the respective temperatures and removed during sampling. Sampling commenced upon addition of IHNV to the seawater samples whereby a 1 ml aliquot was collected to represent the initial infectious virus concentration (denoted as T_0_). Thereafter sampling was performed at various intervals (between 8 and 16 hours) for up to 6 days. To stabilize samples, an antibiotic/antimycotic mixture (Anti-anti; Gibco) was added at 1∶100 to all individual sub-samples and stored at 4°C until analysis by plaque assay which occurred within 4 hours of collection.

**Viral decay in the presence of sunlight.** Aliquots (30 ml) of natural seawater and sterile seawater seeded with 3×10^6^ pfu of IHNV (isolate 93-057) where individually contained in sealed transparent plastic bags. To expose samples to sunlight, the sealed bags were either placed on the water surface of a 2300L outdoor tank or affixed to a rack submersed at 0.5 m depth. As a negative control samples were covered with black bags to eliminate sunlight exposure. In total, 4 treatment groups were tested and are hereafter designated as ‘dark surface; DS’, ‘dark depth; DDp’, ‘light surface; LS’ or ‘light depth; LDp’. Separate bags for each treatment timepoint were placed in the tank such that at designated timepoints individual bags were removed. Prior to sample collection, all samples were shielded from the sun by covering them with a large black bag. To initiate sunlight exposure (T_0_), the plastic bag was removed exposing all ‘light’ samples. For the LS group, samples were removed every 15 minutes while for the LDp group, samples were removed every half hour. The samples for DDp and DS groups were removed every hour. Upon removal of the sample from the tank, each was placed on ice and in the dark and immediately taken to the laboratory for analysis by plaque assay. During each experiment, water flow was turned off in consideration of biosecurity and surface temperature ranged from 12 to 14°C, increasing 2°C over the duration of the experiment. The experiment was carried out in an unshaded area on the grounds of the Pacific Biological Station (Nanaimo, B.C.) on July 27 2010 from 1400 to 1700hrs local time.

**Sunlight measurements.** A major agent for the decay of infectivity of viruses in the environment is solar radiation [17], [18], [19], [20], specifically UVA radiation (200 to 400 nm) and UVB radiation (280 to 320nm). The UVC band (200 – 280 nm) is the most effective at inactivation of viruses but, the oxygen and ozone in the upper atmosphere filter out level almost all of the UVC radiation leaving only UVB & A radiation at ground level [21]. Consequently in the viral decay experiment involving sunlight, UV A and B radiation were monitored with various instruments. Total UVA and UVB were measured underwater with a Solar Light UVA+B detector (PMA2107; Solar Light Co.) and data was collected using a Solar Light PMA2100 data logger. In air, at ground level a Davis Instruments Wireless Vantage Pro 2 (# 6152) weather station fitted with UV and solar radiation sensors measured and recorded UV and solar radiation. The Davis UV sensor (#6490) measured Erythemal UV (280 to 360nm) or the UV Index [22]. The Davis solar radiation senor (#6450) measured total solar radiation from 400 to 110nm. All measurements were conducted through a transparent bag to simulate the sample exposure and measurements were taken at 1 minute intervals.

**Data analysis.** In the viral decay studies without sunlight, the time rate of virus inactivation was modelled as a simple temporal exponential decay (Eq. 1), where *V_0_* is the initial concentration of virus and *V(t)* the concentration at time *t* and *k* is the decay rate constant.



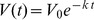
(Eq.1)


Viral decay rate constants were calculated by fitting a least-squares method linear regression to a plot of the natural logarithm of virus concentration (PFU ml^−1^) versus time. The slope of the line is the decay rate constant k (per unit time). The correlation coefficient (R^2^) was calculated to provide information on how well the exponential model fitted the data and 95% confidence intervals were calculated for decay rate constant. Regression analyses excluded data points below 1000 PFU ml^−1^

The degree of inactivation of virus exposed to UV light is dependant on the exposure dosage of UV radiation received by the viral particles [20]. For viral particles exposed to a constant source of UV radiation the exposure dosage is simply the product of exposure time and radiation. For viral particles exposed to time varying UV solar radiation from time *t_1_* to *t_2_*, the UV dosage, *D(t_2_-t_1_)* (J m^−2^) is calculated as the time integral of UV (A&B) radiation, *I_UV_(t)* (W m^−2^) (Eq. 2).



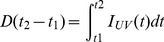
(Eq.2)


The first order the simple exponential decay rate model (Eq.3) quantifies the survival of viral particles exposed to UV radiation dosage *D(t_2_-t_1_),* where *k_UV_* is the decay rate constant (J^−1^ m^2^).



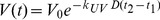
(Eq.3)


### Fish experiments

**Ethics statement.** All fish studies were carried out in accordance with the recommendations in the Canadian Council on Animal Care (CCAC) Guide to the Care and Use of Experimental Animals. The protocols were approved by the Pacific Region Animal Care Committee (Animal Use Protocol Number: 10-001 and 12-027). All fish handling was performed under Aquacalm™ (Syndel Laboratories Ltd) or tricaine methanesulfonate (MS222) anesthesia.

**Fish.** Atlantic salmon (*Salmo salar*) pre-smolts (Mowi strain) reared at a freshwater hatchery on Vancouver Island with no previous occurrence of IHNV were transported to the Pacific Biological Station and held on UV treated 10°C seawater (32 ppt). Fish were fed daily at 1.5% body weight a commercial diet (Ewos). All challenge studies were conducted in 10°C seawater (32 ppt) with Atlantic salmon varying in smolt age and size as specified in Table 1.

**Table 1:** Summary of virus exposure trials.

	Atlantic salmon			Virus		
Challenge Trial	Smolt age	Size (g)	No. per tank	Route	Dose	Duration of experiment (dpc)
Mininum infectious dose	71	123	40	imm.	10^1^ –10^4^	51
Virus shedding						
Exp #1- Population	276	420	88	imm.	4.6×10^3^	51
Exp #2- Individual	215	299	1	i.p.	5.0×10^4^	14
Exp #3- Individual	253	350	1	imm.	4.6×10^3^	35

Virus exposure through intraperitoneal injection (i.p.) or waterbath immersion (imm.) with dose reported as plaque forming units (pfu fish^−1^) and (pfu ml^−1^), respectively. Smolt age represents days after transfer to saltwater and size represents average fish weight at time of challenge. dpc: days post challenge.

**Minimum infectious dose.** To determine the lowest concentration of IHNV necessary to cause mortality in Atlantic salmon smolts, a waterborne immersion challenge was conducted at various doses ranging from 10^1^ – 10^4^ PFU/ml. Treatment groups of 40 fish each (average weight 122 g) where either immersed for one hour in aerated 100 L static seawater bath containing virus or cell culture media (negative control group). Each of the three highest virus doses (10^4^, 10^3^, and 10^2^ PFU/ml) were conducted in triplicate tanks while the lowest dose (10^1^ PFU/ml) was conducted in duplicate tanks. Immediately after addition of virus, a 1 ml water sample was taken to quantify virus dose in each tank. After the one hour immersion challenge, saltwater flow was resumed at a rate of 8L/min with each treatment group containing a fish density of 4.88 kg/m^3^. Fish were monitored daily for mortality and signs of disease for a period of 51 days post challenge where upon all remaining fish were euthanized with an overdose of MS222 (250 mg L^−1^). Virus titers were determined by plaque assay in a minimum of 50% of dead fish from each tank.

Viral shedding rate: Experiment #1-Population level IHNV immersion exposure

Atlantic salmon (n = 90) were acclimatized for 10 days prior to experimental infection in oval tanks containing 2000 L of 10°C sea water at a flow rate of 28 L/min. After the acclimation period, the water flow was stopped and the volume was reduced to 1000L. Virus was added to a final concentration of 4.6×10^3^ PFU/ml and fish were maintained with aeration for 1 hour after which saltwater flow was resumed and the tank was filled to capacity. As a negative (mock) challenge control, fish at an equivalent stocking density were immersion challenge with HBSS.

To determine the shedding profile, two samples of seawater were collected daily from the experimental tank. For water collection, the flow was stopped and fish were maintained with aeration. At 0 and 1 hour, 50 L of seawater was removed from the tank and placed in sterile containers at 4°C. Following the 1 h sample, the water flow was resumed. Water samples were processed immediately or within 48 hours of collection. Samples that were not processed immediately were stabilized with Antibiotic-Antimycotic (1X; Gibco) and stored at 4°C until assayed. Each sample was filtered according to Grant et al (2010) using the FlexStand Cartridge Holder system (Amersham Biosciences) fitted with a hollow-fiber column with a surface area of 1.15 m2 (model number: UFP-500-C-9A; GE Healthcare). Retentate samples were assayed immediately for viable virus using plaque assay as described above.

The viral shedding rates (pfu fish^−1^ hour^−1^) were calculated using the following formula:

Shedding rate  =  (T_1_ waterborne virus titer – T_0_ waterborne virus titer)/fish count

where waterborne virus titer represents the total number of infectious virus partials per tank and was calculated as: waterborne virus titer  =  pfu ml^−1^×volume of tank. Fish count was the total number residing in the tank at time of sampling.

Viral shedding rate: Experiment #2 –Individual Fish virus immersion exposure

To obtain a more precise quantification of viral shedding per individual fish, 10 Atlantic salmon were placed in static water (250 L) water containing 4.6×10^3^ pfu ml^−1^ IHNV and supplemented with aeration for one hour. As a negative control, two fish were immersion exposed to cell culture medium in lieu of virus. After the one hour exposure, individual fish were transferred into separate 40 L tanks containing UV treated 10°C saltwater at a flow rate of 1 L min^−1^. To enumerate shed IHNV, 5 ml samples of seawater were collected on designated days at the beginning (T_0_) and end (T_1_) of a one hour cessation in water flow. Water samples were collected up to and including the day of fish mortality or time of euthanasia which encompassed a maximum of 30 days post injection. Water samples were immediately stabilized by the addition of Antibiotic-Antimycotic (1X; Gibco) and quantified within 48 hours of sampling. Virus concentration in the stabilized water samples was determined using plaque assay on PEG pretreated EPC cells as described above. The shedding rate was calculated using the equation as written above with fish count equating to one for each treatment.

Viral shedding rate: Experiment #3 –Individual Fish virus injection exposure

Atlantic salmon were first anesthesized with MS222 (80 mg L^−1^) and then exposed to IHNV via intraperitoneal (i.p.) injection of 5×10^4^ pfu per fish delivered in a single 100 ul dose. As a negative control, one fish was i.p. injected with virus-free medium. All six fish were housed individually in 4 L tanks containing UV treated 10°C saltwater at a flow rate of 1 L min^−1^. Seawater samples were collected and processed for shed virus titer as described in experiment #2.

## Results

### Virus decay rates without sunlight

**Figure 2:**
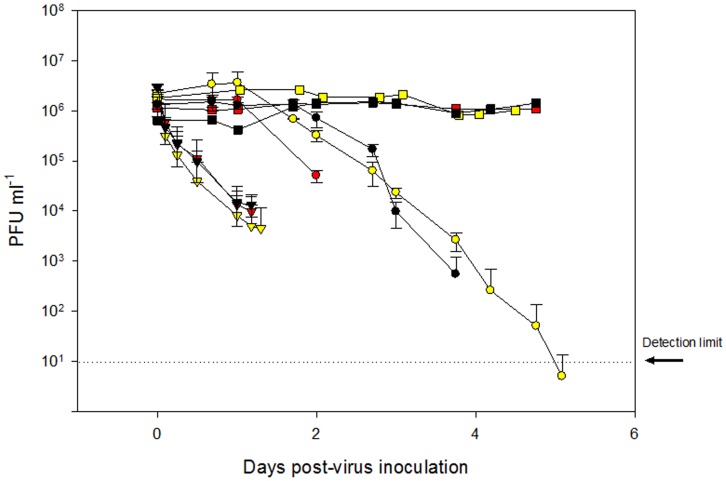
Decay of infectivity of IHN virus in sterile (squares), closed (circles), and open (triangles) seawater systems that were seeded with IHNV and incubated in the dark at 8 (black), 10 (yellow), or 12 (red) degrees Celsius. Error bars represent standard deviation based on triplicate plaque assay counts.

IHN virus decay studies were conducted over various controlled environmental parameters to simulate the range of viral decay rates present in a marine environment. The decay of infectious virus in raw and sterile seawater at three temperatures (8, 10 and 12°C) is shown in Figure 2. Loss of virus infectivity was observed when virus was seeded into a closed or open container of raw seawater while infectivity was maintained when virus was seeded into autoclaved seawater. For the natural seawater samples, the decay rate of the virus was estimated by fitting (least squares) a temporal exponential decay curves (Eq. 1) to the data points at each temperature and for open and closed samples separately.

Table 2 presents the decay rate constants, correlation coefficient R^2^, and 95% confidence intervals for both open and closed samples of natural seawater at three different temperatures. An exponential decay rate provides a good description of the inactivation of the virus in the open containers as is evident by the high R^2^ value (>0.9) for the least square fits. For the samples in closed containers, R^2^ values of the fit are lower and more variable (0.6 to 0.9).

**Table 2:** Least square fit computed decay constants (*k*), 95% confidence intervals and correlation coefficients (R^2^) for IHN virus samples incubated at 8, 10 and 12°C in open and closed containers.

	Open		Closed	
Temperature	*k*±95% C.I. (d^−1^)	R^2^	*k*±95% C.I. (d^−1^)	R^2^
8°C	4.11 ±1.78	0.91	1.35 ±1.21	0.62
10°C	4.18 ±1.39	0.92	1.96 ±0.65	0.90
12°C	4.37 ±1.51	0.94	2.99 ±6.63	0.65

Virus decay appears to be more rapid for the samples incubated in open containers as compared to the samples incubated in containers that were capped to minimize air exchange (Fig. 2). Decay rate constants for open containers were more than twice those for the closed containers (Table 2). However, the overlapping confidence intervals for the 8 and 12°C trials indicate that differences in decay rates are not significant. Only for the 10°C trial are the decay rates of the open samples significantly higher than those of the closed samples. For the open samples, the effect of temperature in the range from 8°C to 12°C on virus inactivation is weak (4.11 to 4.37 d^−1^) and not significant at the 95% level. For those samples incubated in closed containers, the temperature effect on virus inactivation appears to be more pronounced (1.35 to 2.99 d^−1^) than for the samples in open container but the temperature variation in the decay rates for closed samples is not significant given the wide confidence intervals.

### Virus decay rates in sunlight

**Figure 3:**
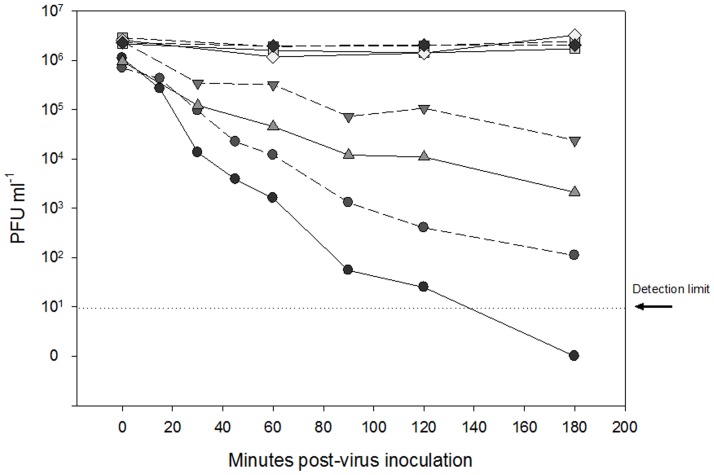
Decay of infectivity of IHN virus in sterile (dashed line) or raw (solid line) seawater while maintained in the dark at depth (0.5 m;squares); in the dark at the surface (diamonds); exposed to sunlight at depth (0.5 m; triangles); exposed to sunlight at the surface (circles).

The decay rate of IHNV was very sensitive to UV radiation. Virus infectivity was reduced by six orders of magnitude for raw seawater samples held at the surface over the 3 hour exposure to direct sunlight while over the same time period infectious virus concentrations remained stable in samples covered with black bags to eliminate sunlight (Dark control) (Fig. 3). A slightly reduced IHNV decay rate was observed when virus was seeded to sterile seawater and exposed to sunlight, suggesting non-UV processes may have contributed towards the inactivation affect.

**Figure 4:**
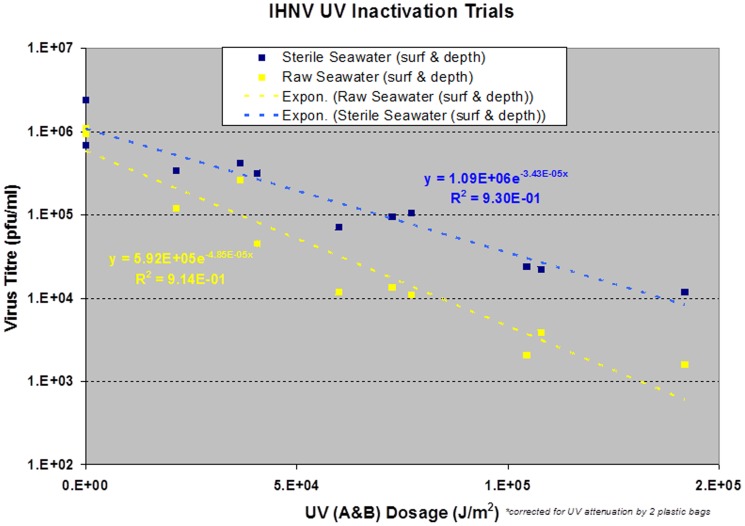
Rate of IHNV decay per UV A and B dosage estimated for raw (yellow) and sterile (blue) seawater samples reported in figure 3. Decay rate of IHNV to exposure of UV radiation calculated via the exponential decay rate model (Eq. 3).

To quantify the effect of UV A&B radiation on virus survival it was first necessary to use the observed UV measurements to compute the UV dosage received by samples held at different depths (surface and 0.5 m depth). For the samples submersed at 0.5 m depth the observations from the Solar Light UVA+B underwater sensor placed next to the samples were integrated to compute UV dosage (Eq. 3). For the samples of virus held at the surface, the Davis Instruments UV sensor data were adjusted and then integrated to compute the UV dosage. It was necessary to adjust the Davis Instruments Erythmal UV sensor measurements because the sensor by design attenuates the UVA radiation (320–400 nm) [22]. The rates of virus inactivation by UV radiation were analysed separately for the raw and sterile seawater groups. Surface and at depth (0.5 m) within each group were combined (Fig. 4).

To quantify the response of the virus to exposure of UV radiation the exponential decay rate model (Eq. 3) was applied, and the UV decay rate was estimated (least squares fit) separately for the raw and sterile seawater sample groups (Fig. 4). The computed decay rates, 95% confidence intervals and correlation coefficient R^2^ for the raw seatwater samples were 4.85×10^−5^ ±1.12×10^−5^ J m^−2^ and 0.91, and 3.43×10^−5^ ±0.71×10^−5^ J m^−2^ and 0.93 for the sterile seawater samples. The correlation coefficient for both groups was high (>0.9) suggesting a good model fit. Although the UV decay rate constant was higher in the raw seawater samples than in the sterile seawater samples, c.f. 4.85×10^−5^ vs 3.43×10^−5^ J m^−2^ the overlapping confidence intervals indicates that the UV decay rates are not significantly different at the 95% confidence level (Fig. 4).

### Minimum infectious dose

**Figure 5:**
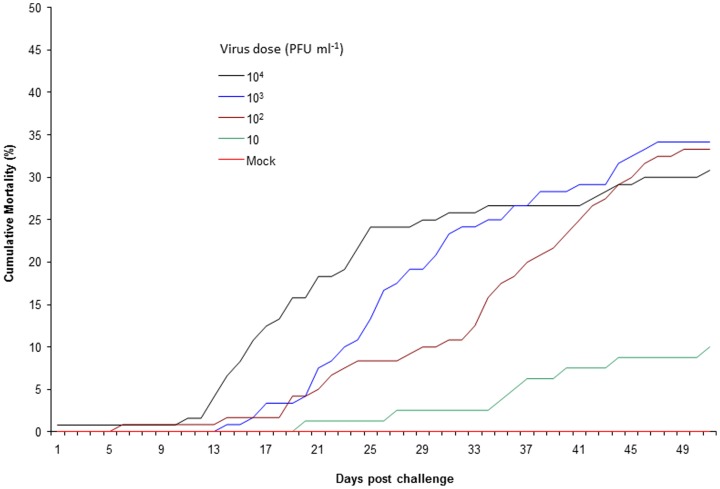
Cumulative mortality of Atlantic salmon waterborne exposed to various doses of IHNV. Data are means from triplicate experiments for doses 10^2^-10^4^ and duplicate experiments for 10^1^ pfu ml^−1^.

Mean exposure titers in the 10^1^, 10^2^, 10^3^, and 10^4^ PFU ml^−1^treatment groups was 18, 240, 2400, and 47,000 PFU ml^−1^, respectively. Discrepancy between calculated dose and observed dose is due to inherent variability of the plaque assay procedure. Among the treatment groups, no mortality was observed in the mock challenge group while mortality was observed at all IHN virus exposure levels tested with cumulative mortality ranging from 17.5 to 50 % (Table 3). Mortality consistently occurred among triplicate tanks for each of the three highest doses (10^4^,10^3^, 10^2^ PFU/ml) while in the lowest virus dose (10 PFU/ml), mortality was only observed in one of the duplicate tanks. Differences in mortality rate were evident between the virus exposure levels, such that both an earlier onset and faster rate of mortality coincided with higher viral dose exposures (Fig. 5). The shortest mean day to death (19 days) occurred at the 10^4^ virus exposure level while longest mean day to death (36 days) was observed at the 10^1^ virus dose (Table 3). Virus was recovered from 94% (73/78) of the mortalities examined. Mean tissue titers in the dead fish ranged from 1.6×10^5^ to 1.1×10^7^ pfu g^−1^ and were similar regardless of challenge virus dose (Table 3).

**Table 3:** Atlantic salmon mortalities and viral tissue titers after waterborne exposure at various IHNV doses.

Virus Dose (pfu ml^−1^)	Tank #	Cumulative Mortality (%)	Mean Day to Death	Mean tissue titer (pfu g^−1^)	Average CPM^a^
10^4^	101	38	22	3.7×10^5^	31±7.6
	102	23	28	3.6×10^5^	
	103	33	19	1.1×10^7^	
10^3^	104	50	27	1.8×10^6^	34±14.0
	105	30	28	5.0×10^5^	
	106	23	28	1.6×10^5^	
10^2^	107	40	33	3.2×10^6^	34±13.7
	108	18	35	2.7×10^6^	
	109	43	31	1.4×10^6^	
10	110	0	0	NA	10±14.1
	111	20	36	8.9×10^5^	

a Cumulative percent mortality and standard deviation.

### IHNV Shedding: Experiment #1

**Figure 6:**
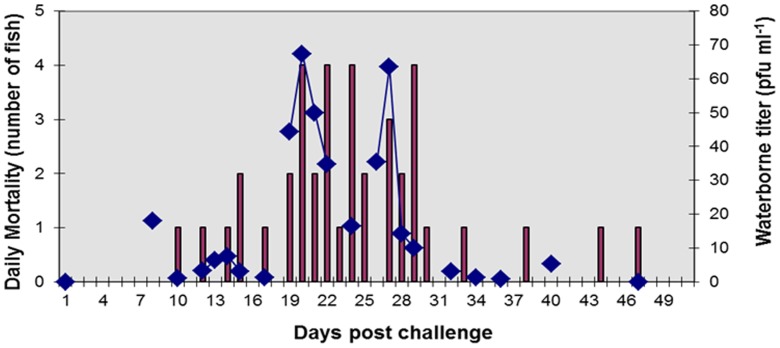
Atlantic salmon waterborne exposed to IHNV. Titer of waterborne IHNV (diamonds) and daily mortality (bars) in a population of Atlantic salmon post smolts exposed to IHNV at a dose of 4.6×10^3^ PFU ml^−1^. Cumulative mortality was 44% upon termination of the experiment 51 days post virus exposure.

An epizootic event was initiated in a laboratory population of Atlantic salmon after a 1 hour immersion exposure to IHNV at a dose of 4.5×10^3^ pfu ml^−1^. Mortality began 10 days post virus exposure with cumulative mortality reaching 44% at 51 days post challenge. No mortality was observed in the tank housing the negative control fish. Viral shedding from infected salmon, monitored through waterborne titers, was detected on the 2^nd^ sampling timepoint at 8 days post challenge (Fig. 6). Viral shedding persisted over the time course of the epizootic with peaks in waterborne titers typically preceding mortality spikes by 1 to 3 days. Viral shedding rates, calculated based on the change of waterborne virus titer during a one hour timeperiod, reached levels of 2.6×10^6^ pfu fish^−1^ day^−1^ at 20 d post exposure (data not shown). It is noteworthy that this maximum shedding rate is likely a conservative estimate as rates per fish assume that all Atlantic salmon in the tank are virus infected and shedding equivalent quantities of virus. Consequently, experiment #2 and #3 were performed to better quantify viral shedding per individual fish as well as assess shedding variability between fish.

### IHNV Shedding: Experiment #2

**Figure 7:**
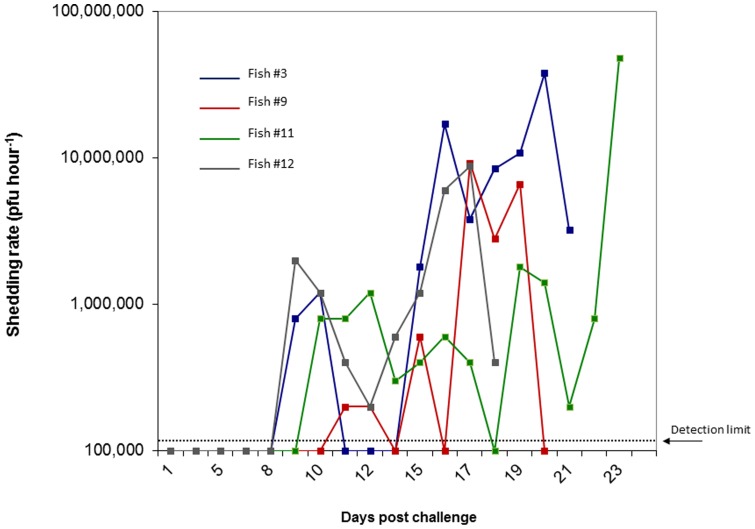
Virus shedding rate of Atlantic salmon waterborne exposed to IHNV. Shed virus concentration calculated based on the change of waterborne virus titer over a one hour timeperiod. Only fish which had measurable levels of shed virus are graphed.

Four of the ten fish (40%), immersion exposed to IHNV and subsequently held in individual tanks, succumbed to mortality or morbidity during a 30 day holding period while no mortality or signs of disease were observed in the negative control fish exposed to media in lieu of virus. Among the virus challenged fish, shed virus was detected in the water of 80% (8/10) of the tanks while no waterborne virus was detected in the negative control tanks. The highest levels of waterborne IHNV were detected in tanks whereupon fish succumbed to mortality (fish #3, 9 and 12) or were euthanized due to extreme morbidity (#11) with peak shedding rates ranging from 8.8×10^6^ to 4.8×10^7^pfu fish^−1^ hour^−1^ (Fig 7). In the tanks where fish mortality occurred within the 30 day holding period (Tank #’s 3, 9 and 12), peak shedding rates preceded mortality by one to two days. In tank #11, peak viral shedding coincided with the day in which the fish from this tank was euthanized due to morbidity. Viral shedding rates were unmeasurable in the remaining six tanks where no fish mortality occurred during the 30 day holding period. Of all fish in the experiment, only the four fish which succumbed to mortality/morbidity during the challenge had positive IHNV tissue titers (> 1.6×10^7^pfu g^−1^) while the remaining virus exposed and negative control fish had no detectable virus titers.

### IHNV Shedding: Experiment #3

**Figure 8:**
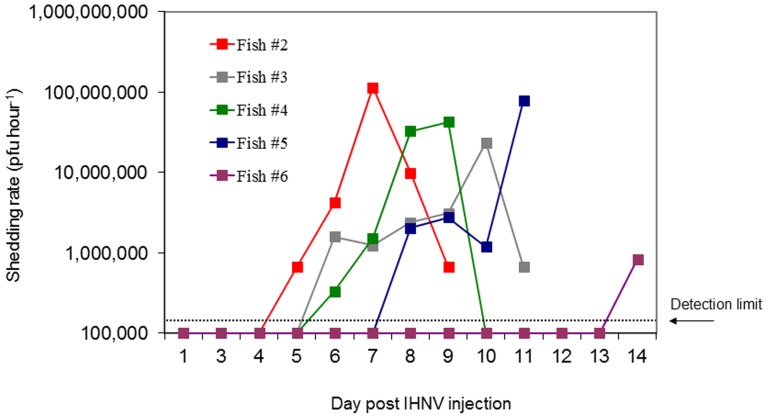
Virus shedding rate of Atlantic salmon injected (i.p) with IHNV. Shed virus concentration (PFU ml^−1^) calculated based on the change of waterborne virus titer over a one hour timeperiod. Only fish which had measurable levels of shed virus are graphed.

Virus challenge via ip injection exposure resulted in 80% (4 out of 5 fish) mortality or extreme morbidity. Shed virus was detected in each of the five tanks housing the virus exposed Atlantic salmon while no virus was detected in the negative control. In tanks whereupon fish succumbed to mortality (#2, 3, and 4) or were euthanized due to extreme morbidity (#5) detectable quantities of virus were present in tank water over six to seven day duration. The largest increase in waterborne virus concentration during a one hour timeperiod (peak virus shedding rate) occurred one (tank 3 and 4) or two (tank 2) days prior to mortality. In tank 5, peak virus shedding was observed on the day of euthanasia. Peak shedding rates for fish #2-5 that exhibited IHN disease signs over the 14 day experiment ranged from 2.3×10^7^ to 1.1×10^8^ pfu fish^−1^ hour^−1^ (Fig. 8). Virus tissue titer in each of the five virus exposed fish was greater than 1.6×10^6^ pfu g^−1^ while no virus was detected in the negative control fish.

## Discussion

This study provides the first quantitative estimates of biological parameters associated with waterborne transmission and dispersion of IHNV within net-pen reared Atlantic salmon. Our results demonstrate that Atlantic salmon smolts are highly susceptible to IHNV, requiring a minimum virus exposure of 10 PFU ml^−1^ for one hour to induce clinical disease and mortality in a population. As host susceptibility to IHNV is undoubtedly influenced by multiple factors including virus strain [23] [24], fish stock [25], species, age, size and lifestage [26], our studies utilized challenge conditions conducive of high infection rates such that minimum infectious dose estimates would be reflective of a worse-case scenario present in an aquaculture setting. To this end, waterborne virus exposures were performed on newly smolted Atlantic salmon (within 71 days transfer to sea water), as it has been noted that a high proportion of farm level outbreaks have occurred in populations that have been in seawater for less than 1 yr [9]. The physiological adaptation to seawater is considered a stressful time whereby immunity to infection is suppressed consequently increasing susceptibility to disease [27]. Moreover, challenge studies herein utilized a virus strain proven to be of high virulence for Atlantic salmon as it originated from an epizootic in farmed Atlantic salmon.

The observation that only 50% of the replicate groups of fish treated with 10 PFU ml^−1^ established IHN disease demonstrates that at this exposure level there is a degree of stochasticity in transmission of virus and that this exposure is near or equal to the minimum infectious dose. Nonetheless, the observation of high viral titers in mortalities from the 10 PFU ml^−1^ exposure group reveals that this low virus dose was sufficient at initiating an epizootic cascade and provides a quantitative estimate of the extreme susceptibility of Atlantic salmon to IHNV. As fish were exposed to higher virus levels, more consistent and rapid infections were established suggesting that virus transmission and infection prevalence was dose-dependent and likely a function of a higher percentage of individuals becoming infected during the period of initial viral exposure. As viral levels exceeded 10^3^ PFU ml^−1^, dose effects were less evident suggesting a threshold challenge level was reached above which little evidence of faster or more severe mortality events where observed. The high level of susceptibility observed in our studies corroborates previous transmission studies which demonstrated Atlantic salmon to be more susceptible to IHNV than sockeye (*Oncorhynchus nerka*), and Chinook salmon (*Oncorhynchus tshawytscha*) [10] and likely reflects their lack of co-evolution with this virus in comparison to the Pacific salmon species which have a long term association with IHNV.

Once infected with IHNV, Atlantic salmon shed increasingly higher quantities of virus with the progression of acute IHN disease. Atlantic salmon succumbing to IHN disease, shed peak levels of virus (geometric mean of 3.2×10^7^ pfu fish^−1^ hour^−1^, Fig. 7 and 8) within a day or two of dying. However such massive shedding rates were only apparent in those fish suffering from acute IHN disease and containing tissue titers while waterborne virus levels were below the lower detection threshold in those tanks containing fish that remained asymptomatic, survived the initial virus exposure, and had unmeasurable tissue titers. This observation reflects the varying capacity within the host population to subvert IHNV infection, replication, and shedding. Consequently viral shedding rates quantified from a population (exp #1) underestimated rates by an order of magnitude (10^6^ vs 10^7^ pfu fish^−1^ hour^−1^) in comparison to those calculated from individual fish (exp #2 & 3) due to the fact that shedding rate calculations presume a 100% infection prevalence in the population when in essence only a small portion of the host population were shedding virus.

By tracking the course of shed virus from individual fish exposed to IHNV, it was revealed that nearly equivalent peak virus shedding rates were reached for each of the 8 Atlantic salmon which succumbed to IHN disease during experiments 2 and 3. The consistency of peak virus shedding rates between experiments suggests that regardless of how the initial acute virus infection is established (either through immersion or injection exposure) the capacity for peak virus amplification remains similar. However differences in the kinetics of virus shedding were observed between experiments 2 and 3, both in the onset and duration of virus shedding. Atlantic salmon waterborne exposed to IHNV (exp #2) exhibited a later onset of virus shedding (an average of 3.5 day later) and shed for a longer duration than fish exposed to IHNV via ip injection (exp #3). These observed differences in virus shedding kinetics are likely consequences of the severity of the initial virus exposure route. As the injection challenge proved to be a more severe challenge than an immersion exposure (83% of Atlantic salmon succumbing to mortality over the 14 day trial versus 40% mortality during a 30 day trial) it is probable that ip injection establishes a more rapid systemic viraemia by circumventing the natural sequential progression of virus to the organs via the circulatory system [28], [29] through the use of mechanical dissemination.

Using the laboratory estimates of IHNV shedding capacity of Atlantic salmon as derived herein, it is possible to quantify virus amplification potential from infected sites. For instance if 1% of 500,000 Atlantic salmon contained in an open net-pen site are undergoing acute IHN disease there is a potential for this outbreak to generate upwards of 1.6×10^11^ pfu in one hour during peak virus shedding. Such a scenario illustrates that if an IHNV epizootic is initiated among high densities of highly susceptible animals that massive quantities of shed virus can result. However to appreciate the role of shed virus in the dispersal and subsequent transmission of IHN disease it is crucial to understand the infectious capacity of the virus when shed from its host.

Virus decay studies presented herein demonstrated that the infectivity of IHNV is markedly reduced over periods of hours when held in natural seawater. The most consistent decay of infectivity is likely biologically mediated as virus decay is more rapid in natural seawater than sterilized seawater (Fig. 2). These results corroborate works of Toranzo et al (1982) which observed a correlation of higher bacterial numbers in waters with greatest viral inactivation and Kamei et al (1987) which demonstrated retention of IHNV infectivity in bacteria-free water versus inactivation by bacterial cultures of *Achromobacter* sp and *Pseudomonas* sp. [30], [31], [32]. Moreover in our studies, it is probable that the slight differences observed between the decay rates of the closed and open systems was a consequence of microbial growth in the sample. Due to the scope of our study, attempts to identify and enumerate the microbial content in water samples were not conducted; nonetheless aeration of the water sample facilitated through an open system, would likely promote an increase in microbial growth as compared to a closed system thereby resulting in the more rapid loss of IHNV infectivity (Fig. 2). As mixing and air exchange are ever present processes in the ocean environment, we speculate that the viral decay constants derived in the open system employed in our laboratory studies are more representative of those occurring in a natural marine environment. Moreover the observation that decay rates are conditional upon multiple factors highlights that a single decay rate cannot be applied universally to all systems.

The infectivity of IHN virus was also significantly reduced via sunlight, specifically UV A and B radiation. Previous work has demonstrated the extreme susceptibility of IHNV to the germicidal effects of UV-C [33], however as this radiation is almost entirely nonexistent at ground level our study focused rather on the impact of solar UV A and B radiation present in a natural environment. When IHNV in seawater was exposed to natural sunlight, infectious virus concentrations were reduced by six orders of magnitude compared to those of dark controls over the 3 hour duration of the experiment. The extreme sensitivity of IHNV to solar radiation has major implications in terms of the persistence of IHNV infectivity in seawater. The relative importance of this affect will vary as a function of the time varying solar UV A and B (seasonally and daily) radiation and water transparency as both UV A and B radiation is attenuated with depth. Based on our observed vertical profiles of UV A and B at several locations in the Discovery Islands area, UV A and B radiation is rapidly attenuated in the water column. For example, at a depth of 7.2 m observed UV A and B was less than 1% of the surface value.

**Figure 9:**
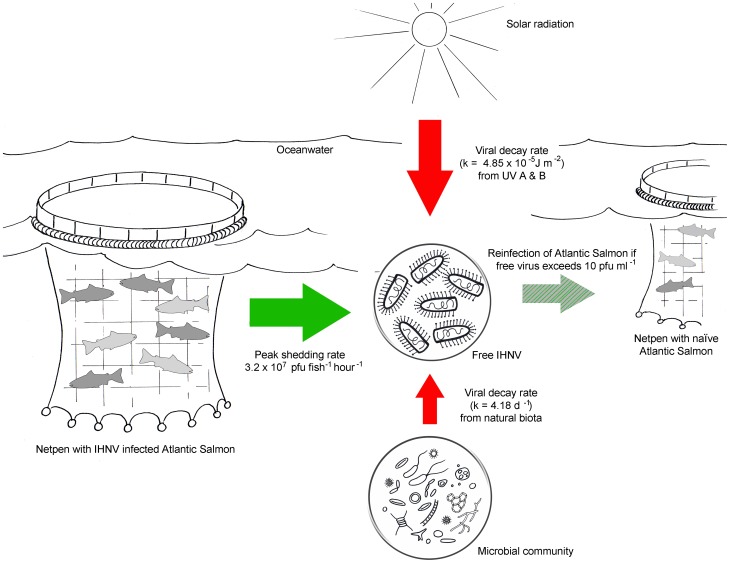
Model depicting the estimated infectious dose, shedding capacity, and decay rates associated with IHN virus transmission among Atlantic salmon marine net-pen aquaculture. Solid arrows represent the production (green) and destruction (red) of viral particles in a 10°C seawater environment while the hatched arrow represents viral transmission if free virus exceeds the minimum infectious dose.

As IHN virus is a significant threat to both cultured and wild salmonid fish, either through hindering enhancement efforts of endangered species or causing significant economic losses within aquaculture operations, there is a need to understand the processes controlling the abundance, infectivity, and spread of this virus. When applied collectively, the quantitative estimates determined in our study form the basis of a simple virus transmission model (Fig. 9) which illustrates that when an IHNV epizootic is initiated, a high viral abundance is generated with the fate of this free virus being tightly regulated by solar radiation and the aquatic microbial community. If the epizootic event permits the production of free virus to supersede the viral destruction rate then transmission of an infectious dose becomes a risk. However to quantify the risk of an infectious dose being dispersed requires knowledge of water movement in the environment of interest. As the overarching goal of our studies are to better understand propagule dispersal from aquaculture facilities, significant efforts have been devoted towards the development of an ocean circulation model for the Discovery Islands, British Columbia [34], an area where numerous Atlantic salmon farms reside (Fig 1). Through coupling the biological estimates determined empirically herein with the physical water circulation model, accurate geospatial predictions of risk for IHNV transmission will be possible.
